# CircRNA-protein complexes: IMP3 protein component defines subfamily of circRNPs

**DOI:** 10.1038/srep31313

**Published:** 2016-08-11

**Authors:** Tim Schneider, Lee-Hsueh Hung, Silke Schreiner, Stefan Starke, Heinrich Eckhof, Oliver Rossbach, Stefan Reich, Jan Medenbach, Albrecht Bindereif

**Affiliations:** 1 Institute of Biochemistry, University of Giessen, Heinrich-Buff-Ring 17, D-35392 Giessen, Germany; 2 Biochemistry I, University of Regensburg, Universitätsstrasse 31, D-93053 Regensburg, Germany

## Abstract

Circular RNAs (circRNAs) constitute a new class of noncoding RNAs in higher eukaryotes generated from pre-mRNAs by alternative splicing. Here we investigated in mammalian cells the association of circRNAs with proteins. Using glycerol gradient centrifugation, we characterized in cell lysates circRNA-protein complexes (circRNPs) of distinct sizes. By polysome-gradient fractionation we found no evidence for efficient translation of a set of abundant circRNAs in HeLa cells. To identify circRNPs with a specific protein component, we focused on IMP3 (IGF2BP3, insulin-like growth factor 2 binding protein 3), a known tumor marker and RNA-binding protein. Combining RNA-seq analysis of IMP3-co-immunoprecipitated RNA and filtering for circular-junction reads identified a set of IMP3-associated circRNAs, which were validated and characterized. In sum, our data suggest that specific circRNP families exist defined by a common protein component. In addition, this provides a general approach to identify circRNPs with a given protein component.

Circular RNAs (circRNAs) are a new and large class of noncoding RNAs comprised of hundreds to thousands of members, and present in all eukaryotes investigated so far, from protozoa, yeast to human^1^,^2^. Although single examples of circular RNAs had been known for decades, starting with the plant-pathogenic viroid RNAs^3^, only in 2012/13 circRNAs were rediscovered as a new class of noncoding RNAs^4^,^5^,^6^. This was based on RNA-seq and corresponding bioinformatic analyses, relying on the circRNA-characteristic splice junction (“back-splice”).

This type of circRNAs contains one or more adjacent exons, that are spliced out of the pre-mRNA to yield instead of the normal, linear protein-coding mRNA a circular RNA product. CircRNAs are cell-type specifically expressed, more stable than the corresponding linear mRNA, evolutionarily conserved, and mostly cytoplasmic in cellular localization^1^. Often circularizing exons are flanked by long introns, which contain reverse-complementary repeats, in human frequently Alu repeats; this presumably allows a cross-exon base-pairing interaction, making circularization more favorable^5^,^7^,^8^,^9^,^10^. We have recently demonstrated by mutational analysis that circularization requires the canonical splice signals, including branch point and pyrimidine tract, arguing for the normal spliceosomal machinery and a mode of alterntative splicing to function here^9^. Exon circularization competes with normal linear splicing, and it is largely unknown what governs the ratio circular-versus-linear product, except for the case of the MBNL circRNA, where the protein product autoregulates its own circular/linear processing pathways^11^. In addition, the splicing regulator protein QKI can control circRNA formation, by binding to intronic QKI sites flanking the circularizing exon^12^. Note that besides these exonic-type circRNAs, there is also a class of intron-derived circular RNA species^13^.

Functionally, circRNAs are still largely uncharacterized, except for a miRNA sponge function, which has been experimentally validated for two cases, ciRS-7 and SRY circRNAs^6^,^14^, and an autoregulatory potential demonstrated for MBNL expression in *Drosophila*^11^. Several other, hypothetical roles have been discussed, for example in protein sponging, allostery, complex assembly, or antisense activity^1^,^15^. Translation of circRNAs to peptides or proteins represents another potential biological function, and earlier studies had demonstrated that translation from circular RNA constructs is in principle possible, for example by inserting viral internal ribosome entry sites (IRES) or under *in vitro* conditions^16^,^17^. However, no natural examples of circRNA translation have been reported so far.

Here we describe the initial characterization of circRNA-protein complexes (circRNPs). For a set of relatively abundant circRNAs we demonstrate that they exist in the form of discrete RNPs, stable in sedimentation analysis through glycerol gradient centrifugation. We then focus on IMP3 (=IGF2BP3, insulin-like growth factor 2 binding protein 3), a known oncofetal and tumor marker RNA-binding protein with multiple post-transcriptional roles^18^,^19^,^20^. In particular, there is evidence for IMP3 playing a role in pancreas development, with IMP3 being overexpressed in pancreatic ductal adenocarcinomas^18^,^21^. Here we combined immunoprecipitation, RNA-seq, and bioinformatic circular-junction analysis, to identify a subfamily of circRNAs stably associated with IMP3, followed by validation and further characterization of several examples of IMP3-containing circRNPs. This provides a general approach to identify circRNPs carrying a specific protein component. Our data indicate that specific proteins define subclasses of circRNPs possibly linked by a common function or biogenesis pathway.

## Results and Discussion

### Evidence for distinct cytoplasmic circRNPs in mammalian cells

**Figure 1:**
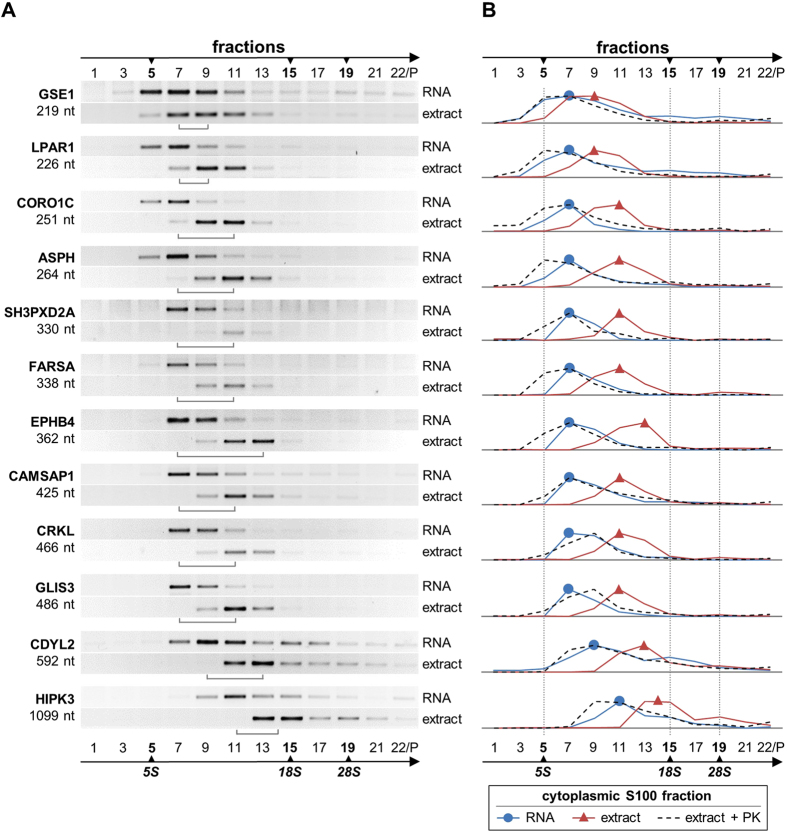
Evidence for distinct circRNA-protein complexes in mammalian cells. (**A**) Sedimentation profiles of circRNPs/circRNAs. Cytoplasmic S100 extract and corresponding free RNA from HeLa cells were fractionated by glycerol gradient centrifugation (#1–22 from top to bottom; the last fraction containing the resuspended pellet), followed by RT-PCR analysis of 12 abundant circRNAs across the gradient (ordered from top to bottom according to their sizes; given in nucleotides). The positions of ribosomal RNA size markers are indicated (5S, 18S, and 28S), as well as the shift of the circRNA vs. circRNP peak fractions (brackets). (**B**) Quantitation of circRNA distribution across gradient, comparing free RNA prepared from extract (RNA, in blue), cytoplasmic S100 fraction (extract, in red), and proteinase K-treated extract (extract + PK, in dashed lines). Data from panel A (RNA/extract) and from Supplementary Fig. S1A (extract + PK) were used for quantitation.

To identify circRNA-protein complexes, we subjected cytoplasmic extract (S100) from HeLa cells to glycerol gradient fractionation, whereby RNAs and RNPs sediment according to their molecular size and shape. We focussed the analysis on S100 extract, since circRNAs predominantly localize in the cytoplasm. 22 fractions were collected, followed by RNA preparation from every second fraction and RT-PCR assays for 12 relatively abundant circRNAs, based on circular-junction-specific primers. In parallel, and for direct comparison, total RNA prepared from S100 extract was fractionated through glycerol gradients under the same conditions, followed by the RT-PCR analysis of the circRNA distribution across the gradient (Fig. 1A; for quantitation of these results, see Fig. 1B).

The free circRNAs distribute in the glycerol gradient between fractions #7 and #11, depending on the size of the respective circRNA (219 nts for GSE1 to 1,099 nts for HIPK3). In contrast, in the S100 extract the circRNAs peak at fractions #9 to #14, but in most cases with a clear peak, indicating that each circRNA exists in the form of a major distinct large complex in the 10–15 S region. To obtain direct evidence that this is due to protein components, we also analyzed the gradient distribution of circRNAs after proteinase K treatment: Clearly, the circRNAs shifted back to the position of free RNA (Fig. 1B and Supplementary Fig. S1), demonstrating that the large complexes we detect in S100 extract represent circRNPs. The difference between the positions of free circRNAs and respective circRNPs are between two fractions (for the small circRNAs, such as GSE1 or LPAR1) and four fractions (for the larger circRNAs, such as GLIS3 or CDYL2); this shift corresponds to a molecular mass difference of approximately 50 to 110 kDa (two to four fractions).

We have also used the same approach to identify circRNP complexes in a cytoplasmic extract prepared under less stringent conditions than S100 (without the centrifugation step at 100,000 × *g*), as well as in nuclear extract (Supplementary Fig. S1): CircRNPs detected in cytoplasmic extract were generally larger than the corresponding complexes from S100 extract (differing by two to four fractions), and they were more heterogeneous in gradient sedimentation, but also protein-dependent; circRNPs in nuclear extract, as far as they were detectable (see CAMSAP1, GLIS3, and HIPK3), behaved similarly as in cytoplasmic extract.

In sum, our analysis provided biochemical evidence for that each of the circRNAs tested exists in cells as circRNPs of distinct sizes.

### Selected abundant circRNAs are not associated with polysomes

**Figure 2:**
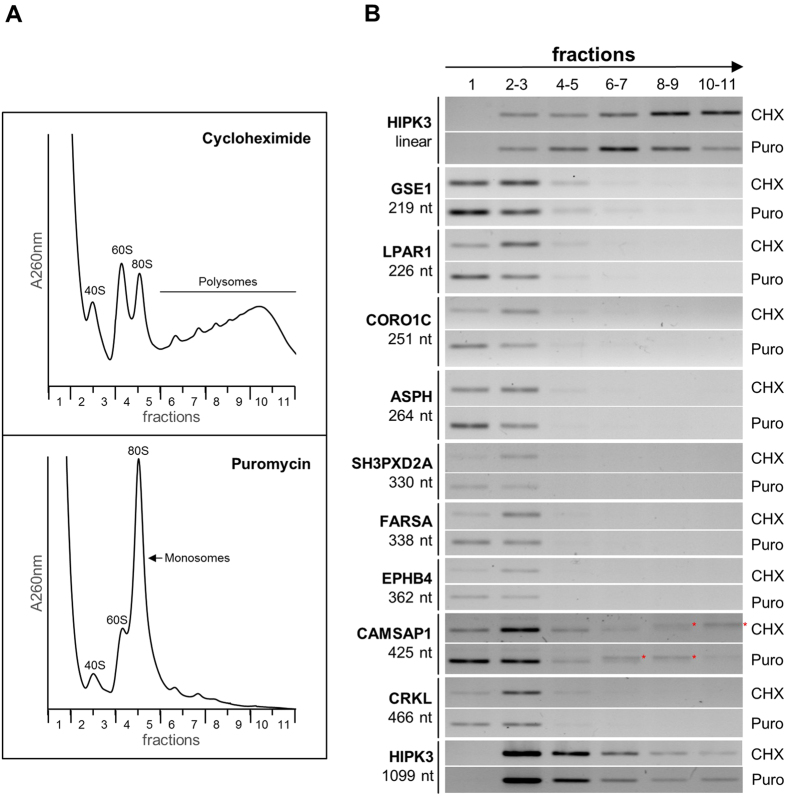
Major circRNAs in HeLa cells are not associated with polysomes. (**A**) Polysome gradient profiles of HeLa cytoplasmic extracts after treatment with cycloheximide or puromycin. The RNA distribution (A_260nm_) and the positions of ribosomal size markers are indicated (40S/60S ribosomal subunits, 80S initiation complex/monosomes, and polysome region). (**B**) Analysis of circRNA fractionation on polysome gradients, comparing profiles after cycloheximide (CHX) or puromycin (Puro) treatment. After RNA extraction, fractions were pooled (#1, #2–3, #4–5, #6–7, #8–9, and #10–11), followed by RT-PCR analysis of ten abundant circRNAs across the gradient. HIPK3 linear mRNA was used as a positive control for puromycin-induced mRNA release from polysomes (top two panels); the ten circRNAs (from GSE1, 219 nt, to HIPK3, 1099 nt) are ordered according to their sizes. Mispriming products corresponding to the linear mRNA of CAMSAP1 in polysome fractions (CAMSAP1 circ, #6–11) are marked by asterisks.

To analyze whether endogenous circRNAs are associated with polysomes and may be translated in HeLa cells, we selected 10 out of the 12 abundant circRNAs described above. Cells were first treated with cycloheximide to stabilize the RNA-ribosome interaction, or, as an important control, with puromycin, which releases ribosomes from mRNA being translated (Fig. 2A). Cytoplasmic extracts were prepared, loaded onto 10–50% sucrose gradients, and subjected to ultracentrifugation. Following fractionation, the distribution of linear HIPK3 mRNA (as a positive control) and of 10 abundant circRNAs across the gradient was determined by RT-PCR (Fig. 2B). As expected, we detected for the linear HIPK3 mRNA a characteristic shift upon puromycin treatment, indicative of translational activity (Fig. 2B, HIPK3 linear mRNA, from fractions #10–11 to 6–7). In contrast, the 10 circular RNAs analyzed showed a similar sedimentation across the gradient, with peaks in fractions #1–3 (that is up to the 40S region), and with only very minor quantities (if detectable at all) in the monosome-to-polysome fractions (#4–11). Importantly, we did not observe any significant shift of these cirRNAs upon puromycin treatment (compare gradient distributions CHX versus Puro). Only for HIPK3 we found considerable quantities of circRNPs in the polysome region, yet without any major change upon puromycin treatment. Note that for CAMSAP1 circRNA we observed two RT-PCR products of very similar sizes (see fractions #6–11): The bottom band represents the circular RNA; the top band (marked with an asterisk) is due to mispriming at the linear CAMSAP1 mRNA and shifts upon puromycin treatment, as expected from translationally active linear mRNA.

We conclude that our polysome gradient analysis did not yield any evidence for circRNAs to be efficiently translated, as shown here for 10 abundant circRNAs in HeLa cells. This is consistent with three other recent reports, based on ribosome-footprinting and polysome-gradient data from mammalian cell lines^5^,^22^ and mouse brain^23^. Although our carefully controlled initial analysis argues against a widespread translational potential of circRNAs, obviously this does not exclude that there may be natural cases of circRNA translation, for example restricted to a small subset of specialized circRNAs, or to certain cell types, tissues, developmental stages, or growth conditions.

### Identification of IMP3-associated circRNAs

To characterize circRNPs further, we initially tried to use the iCLIP approach (individual-nucleotide crosslinking-immunoprecipitation), which allows mapping of RNA-protein contacts at single-nucleotide resolution^24^. Because of the initial immunoprecipitation (IP) step, this approach had to focus on a specific protein to be characterized as a potential circRNP component. We chose IMP3 (also called IGF2BP3, insulin-like growth-factor 2 binding protein 3), a known multifunctional RNA-binding protein implicated in posttranscriptional gene regulation and an established tumor marker protein (see *Introduction*). We mapped transcriptome-wide binding sites for IMP3 protein by iCLIP in HepG2 cells (human liver carcinoma cell line), followed by searching for iCLIP tags spanning the characteristic circular junctions (see Supplementary Fig. S2 for the initial IMP3 iCLIP analysis and specific examples of IMP3 targets). However, it turned out that due to short sequencing reads and limited sequencing depth, our analysis of these IMP3-iCLIP data was not suitable for circRNA identification.

**Figure 3:**
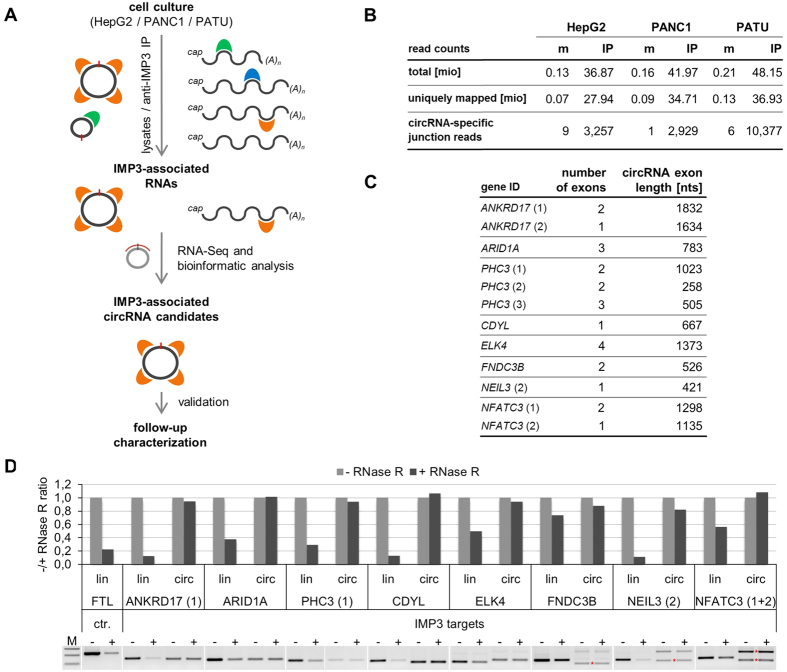
Identification of IMP3-associated circRNAs: anti-IMP3 co-immunoprecipitation and RNA-seq. (**A**) Strategy of identification of IMP3-associated circRNAs. For details, see *Results*. (**B**) Summary of RNA-seq analysis, based on immunoprecipitates from three different cell lines (HepG2, PANC1, and PATU), for each case comparing mock control (m) and after IMP3 immunoprecipitation (IP). Total and uniquely mapped read counts (each in mio reads), as well as circRNA specific junction reads are listed. (**C**) Candidate list of 12 IMP3-associated circRNAs, including number of circularized exons and their total length (in nts). For three circRNAs, different circRNA isoforms (as indicated) were identified to be IMP3-associated. (**D**) Evidence for circularity of IMP3-associated circRNAs. Total RNA from HepG2 cells was mock- or RNase R-treated (−/+), followed by RT-PCR detection of circular and linear isoforms of IMP3 targets. The linear mRNA of FTL served as a positive control for RNase R digestion. M, markers (100, 200, and 300 bp). Results are shown after agarose gel electrophoresis (bottom) and by quantitation (top; −/+ RNase R ratio), normalized to the mock-treated sample. In lanes with multiple PCR products the band specific for the respective circRNA is marked by an asterisk.

Therefore we developed an alternative approach, combining IP, RNA-seq, and bioinformatic filtering for circRNA junction sequences (Fig. 3A).

First, cell lysates were prepared from cultured HepG2 cells, in parallel also from PANC1 and PATU cells, two human pancreas tumor-derived cell lines. All of these three cell lines express IMP3 protein (data not shown).

Second, IMP3-associated RNAs were obtained by anti-IMP3 immunoprecipitation, RNA-seq library construction, and sequencing (~127.5 mio. 2 × 100 bp paired-end reads, Illumina HiSeq 2000). The major part of the total sequenced reads (~127.5 mio.) was from the IMP3-immunoprecipitated material (~36.9/42.0/48.2 mio. reads for the respective HepG2/PANC1/PATU libraries; Fig. 3B); total read numbers from the control immunoprecipitations were below 0.5%.

Third, IMP3-associated circRNAs were predicted by extracting circRNA-specific junction reads, resulting in 3,257/2,929/10,377 reads for the respective HepG2/PANC1/PATU libraries (Fig. 3B). From the ~5,000 putative circRNAs, a set of 34 circRNAs containing exons from 25 protein-coding genes were considered with high confidence to be IMP3-associated (≥30 circRNA-specific junction reads in at least one of the 3 cell lines; see Supplementary Table S1). For further experimental analysis, 8 different circRNAs with high circRNA-specific junction read counts in all three cell lines were selected (Fig. 3C, D).

As evidence for the circularity of these putative IMP3 target circRNAs, we assayed their resistance towards RNase R, an exo-ribonuclease, using RT-PCR specific for the circular versus a downstream linear splice junction: All circRNA candidates selected showed high RNase R resistance, relative to the corresponding linear isoform from the same gene (Fig. 3D).

### An IMP3-associated family of circRNPs: validation, characterization, and specific IMP3-circRNA binding

**Figure 4:**
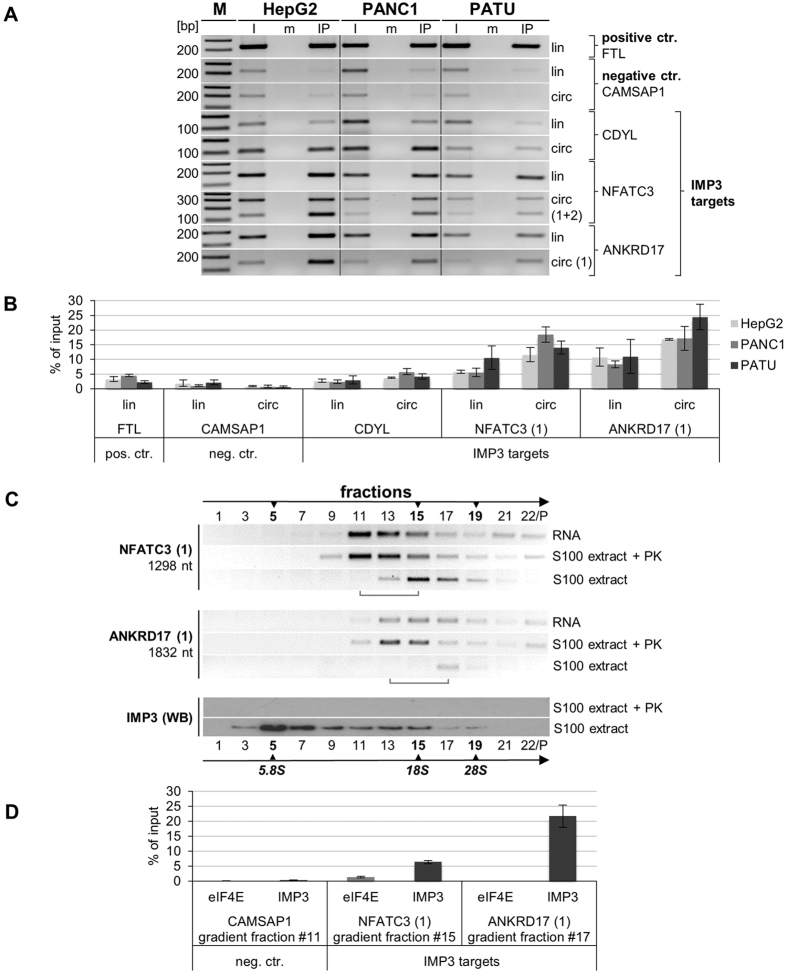
IMP3 protein specifically and stably bound to circRNA family: validation and characterization. (**A**) Validation of IMP3 bound to specific circRNAs. Lysates of HepG2, PANC1, and PATU cells were subjected to immunoprecipitation with anti-IMP3 antibodies (IP), or as mock control, with anti-FLAG antibodies (m). Co-precipitated RNA was purified and assayed by RT-PCR for FTL mRNA (positive control), CAMSAP1 (negative control circRNA), and for the putative IMP3-associated circRNAs CDYL, NFATC3, and ANKRD17. For each of the circRNAs, the linear isoform of the respective gene was tested in addition (circ/lin; 90% of the mock- and IMP3-immunoprecipitates were used in RT-PCR). In addition, 5% of the input material was assayed (I). M, markers (in bp). (**B**) Quantitative immunoprecipitation analysis of IMP3-circRNA association in three cell lines (HepG2, PANC1, and PATU). For the same set of IMP3 circRNA targets and controls as shown in panel A, the immunoprecipitation efficiences were determined by RT-qPCR assays (% of input; statistical deviations based on biological duplicates). (**C**) Sedimentation profiles of IMP3-containing circRNPs. Cytoplasmic S100 extract from HeLa cells (extract), corresponding free RNA (RNA), and proteinase K-treated extract (extract + PK) were fractionated by glycerol gradient centrifugation (#1–22; the last fraction contains the resuspended pellet), followed by RT-PCR analysis of two IMP3-containing circRNAs (NFATC3 and ANKRD17). The relatively low recovery of circRNAs NFATC3 (1298 nt) and especially ANKRD17 (1832 nt), the largest circRNAs analyzed in S100 extract, may be caused by the higher tendency of such large circRNPs to aggregate and form precipitates, which were lost in the pellet fraction. The positions of ribosomal RNA size markers are indicated (5S, 18S, and 28S), as well as the shift of the circRNA vs. circRNP peak fractions (brackets). For comparison, the distribution of total IMP3 protein across the gradient was visualized by Western blotting in extract and, as a control, in proteinase K-treated extract. (**D**) IMP3 immunoprecipitation efficiencies of gradient-purified NFATC3 and ANKRD17 circRNPs. HeLa cytoplasmic S100 extract was gradient-fractionated, and NFATC3 and ANKRD17 circRNPs were IMP3-immunoprecipitated from the respective peak fractions (NFATC3, #15; ANKRD17, #17), using CAMSAP1 circRNA (peak fraction #11) and anti-eIF4E immunoprecipitation as negative controls. Immunoprecipitation efficiencies (% of input; statistical deviations based on technical triplicates) were determined by RT-qPCR.

To validate and quantitatively characterize the IMP3 association of a subgroup of circRNAs we did anti-IMP3 immunoprecipitations from lysates of HepG2, PANC1, and PATU cells, followed by RNA purification and semiquantitative RT-PCR assays of circular and linear isoforms for three IMP3 circRNP candidates (CDYL, NFATC3, and ANKRD17), comparing in each case input (5%) and immunoprecipitate (90%); as a negative IP control, anti-FLAG IPs were analyzed in parallel (Fig. 4A). As another negative control, both linear and circular variants of CAMSAP1 were analyzed; the linear FTL mRNA, which we knew from our iCLIP study to be IMP3-associated (see above and Supplementary Fig. S2B), served as a positive control.

As a result, we were able to confirm all three IMP3 circRNP candidates as IMP3 targets (CDYL, NFATC3, and ANKRD17); both linear and circular isoforms of CAMSAP1, in contrast, exhibited only background or undetectable levels after IMP3 immunoprecipitation. These semiquantitative results were further confirmed by real-time PCR assays (Fig. 4B): The anti-IMP3 IP efficiencies were 4–5% for CDYL, 11–18% for NFATC3, and 16–24% for ANKRD17 circRNA, with only minor differences between the three cell lines.

As the next step, we fractionated HeLa cytoplasmic extract (S100) by glycerol gradient centrifugation, in parallel with total RNA prepared from the same extract, and proteinase K-treated S100 extract (Fig. 4C). The two major IMP3-associated circRNAs, NFATC3, and ANKRD17, were detected in the gradient fractions by RT-PCR, using circular-junction-specific primers, analogously to the characterization of abundant circRNAs (see above and Fig. 1). Both IMP3-associated circRNAs showed a distinct peak, suggesting that a predominant complex exists, which concentrated in fractions #15 (NFATC3) and #17 (ANKRD17). Corresponding free circRNAs sedimented approximately four fractions more towards the top, and very similarly as deproteinized circRNPs, confirming that the circRNA complexes contain protein components. We noted that the free circRNAs for NFATC3 (1298 nt) and ANKRD17 (1832 nt) show a broader distribution across the gradient (Fig. 4C), similarly as the free circRNAs of CDYL2 (592 nt) and HIPK3 (1099 nt; Fig. 1), compared with smaller circRNAs. This may reflect the higher potential of large circRNAs to form alternative structures (or aggregates by RNA-RNA interactions) that would affect their sedimentation behavior. The distribution of IMP3 protein was detected by Western blotting in the same gradient fractions: A large portion of IMP3 protein peaked in fraction #5, most likely representing free protein, but the rest of it distributed across fractions #7–19, that includes the region where the IMP3 circRNPs fractionated.

Finally, we measured IMP3 association for two of these gradient-purified circRNPs, NFATC3 and ANKRD17, using anti-IMP3 immunoprecipitation from the respective peak fractions (NFATC3, #15; ANKRD17, #17), followed by real-time RT-PCR for the circular splice junctions; as negative controls we used IP against eIF4E, an abundant cytoplasmic RNA-binding protein, which binds to the cap structure of linear mRNAs, and CAMSAP1 circRNA, which was not among the IMP3 targets identified (Fig. 4D). The IP efficiencies of the NFATC3 and ANKRD17 IMP3 circRNPs with IMP3 antibodies were 6.4 and 21.7%, respectively, background binding below 1.3% (anti-eIF4E IP) or undetectable (for CAMSAP1 circRNP). In sum, we thereby confirmed that IMP3-containing circRNPs of the NFATC3 and ANKRD17 circRNAs exist and are stable through glycerol gradient centrifugation.

### Specificity of IMP3-circRNA binding based on a SELEX-derived C/A-rich motif

**Figure 5:**
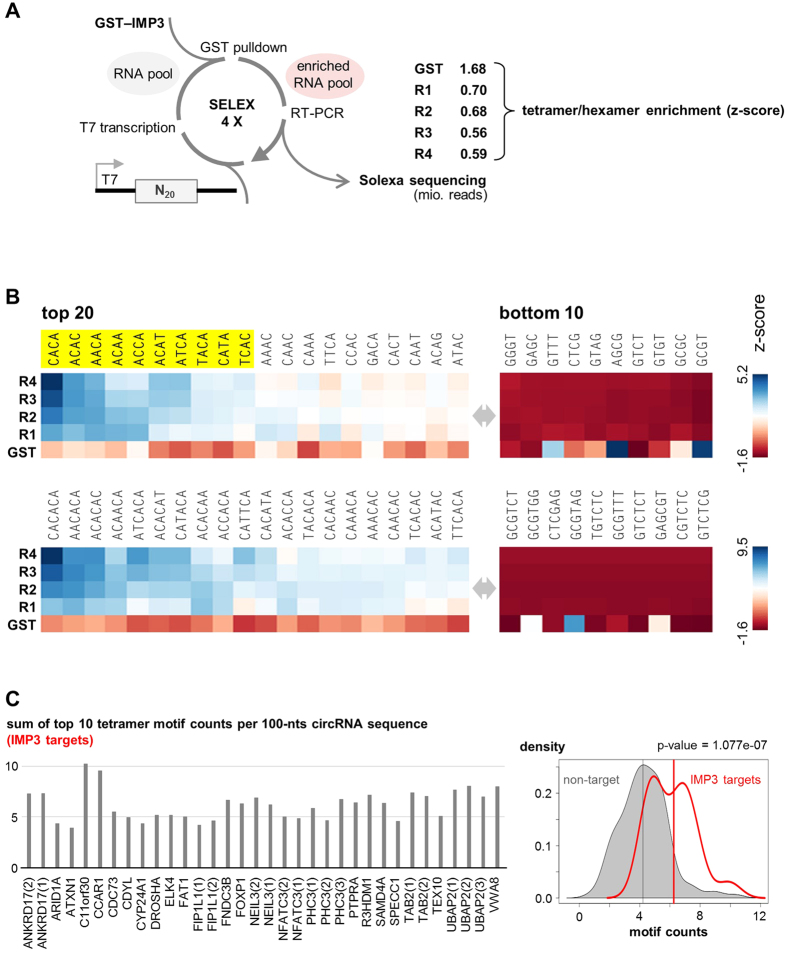
IMP3 binding motif derived from SELEX-seq analysis: enrichment in IMP3-associated circRNAs. (**A**) Schematic representation of SELEX-seq procedure and data analysis. Using GST-IMP3 (GST as control) and an N_20_ RNA pool, IMP3 RNA-binding sequences were enriched through four SELEX rounds. After each round (R1–R4) and for the GST control, aliquots were taken for Solexa sequencing (number of reads given in mio), followed by motif enrichment analysis (see panel B). (**B**) Tetramer and hexamer enrichment for the four SELEX rounds with GST-IMP3 (R1–R4) and GST control (only R4). Only the top 20 and the bottom 10 tetramers/hexamers are represented as colour-coded heat map, showing z-scores of the motif frequencies for each round. Motifs are ordered according to their cumulative (R1–R4) z-score. The top 10 tetramer motifs (highlighted in yellow) were used for motif enrichment analysis of specific circRNAs (see panel C). (**C**) IMP3-binding motif enrichment in IMP3-associated circRNAs. The sum of the top ten tetramer motif counts in each of the IMP3 target circRNAs was determined (left part) and compared with a non-target group of circRNAs (n = 117). This is represented by a kernel density estimation plot with group median values shown by vertical lines (p-value 1.077e-07; Welch two sample t-test; right part).

To explain the specific and stable IMP3 association with a subset of circRNAs, we further investigated the intrinsic RNA-binding specificity of the IMP3 protein. We therefore applied an *in vitro* SELEX procedure, using four rounds of selection with an N_20_ RNA pool and recombinant GST-IMP3, which contains the full-length IMP3 protein (Fig. 5A). GST protein served as a control in parallel selection and enrichment rounds. After each round, aliquots of the RT-PCR amplified RNA pools were analyzed by Solexa sequencing, resulting in between 0.56 and 0.70 mio sequence tags per cycle for GST-IMP3 (for the GST control: 1.68 mio tags after the fourth cycle).

The representation of each of the 256 possible tetramer motifs was determined after each SELEX cycle and the enrichment of each tetramer evaluated by z-score values. Figure 5B shows as a heatmap only the top 20 tetramers (ordered according to their z-score sum of the four SELEX rounds with GST-IMP3 protein) and the bottom 10 tetramers, in particular how these motifs changed over the four cycles (R1 to R4). Clearly, the top 10 tetramers (highlighted in yellow) are highly C/A-rich, with CACA, ACAC, and AACA becoming enriched most strongly, consistent with the “compendium motif” of Ray *et al*.^26^. On the other extreme, the bottom 10 motifs are all G/C- or G/T-rich. A corresponding analysis of hexamer motif enrichment confirmed these results (Fig. 5B).

To analyze whether these SELEX-based IMP3 RNA-binding motifs are enriched in the IMP3-associated circRNAs, we calculated the sum of the top 10 tetramer motif counts in 34 IMP3-bound circRNAs, normalized to 100 nts sequence length (Fig. 5C, left part). These motif counts in this IMP3 target circRNA group were compared with those in a non-target group (circRNAs well-expressed in HepG2 cells, n = 117; for details, see *Methods*). Based on kernel density estimation and a p-value of 1.077e-07 (Welch two sample t-test), we conclude that C/A-rich motifs are significantly enriched in IMP3-associated circRNAs (Fig. 5C, right part).

In conclusion, the IMP3 protein by itself can recognize certain C/A-rich motifs. The specificity of IMP3 RNA recognition is most likely complex, due to its domain structure with two RNA-recognition (RRMs) and four KH motifs. This may contribute to why the motif from a PAR-CLIP-based *in vivo* study in HEK293 cells (CAUU)^25^ differed from an *in vitro* analysis (CA-rich motif)^26^. There may also be additional factors, for example associated other RNA-binding proteins, that modulate the RNA-binding preference of IMP3 *in vivo* or act in a combinatorial manner. Therefore we have to consider that IMP3 may bind in 3′-UTR versus coding-exon regions through different ways and with different cofactors, reflected in different enriched motifs.

## Methods

### Glycerol gradient sedimentation analysis

Cytoplasmic S100 fraction (**S100**), cytoplasmic extract (**CE**; prepared without the centrifugation step at 100,000 × *g*), and nuclear extract (**NE**) from HeLa cells (IpraCell), as well as RNA isolated from S100 extract (**RNA**), proteinase K-treated S100 (**S100** + **PK**), and proteinase K-treated cytoplasmic extract (**CE** + **PK**) were analyzed by glycerol gradient centrifugation. For the gradient with free RNA, RNA from 500 μl S100 was isolated by TRIzol (Ambion). Proteinase K (PK) treatment was done by incubating 500 μl extract (**S100/CE**) in 1x PK buffer with 100 μg/ml PK (Roth) and 0.5% SDS for one hour at 37 °C. Samples of 500 μl each were loaded onto 10–30% (v/v) glycerol gradients (10 ml) and subjected to ultracentrifugation for 16 hours at 4 °C (32,000 rpm; SW-40). After centrifugation, the gradient was fractionated manually into 21 fractions of 500 μl each, whereas the resuspended pellet was labeled as fraction 22. RNA was isolated from 200 μl of every second fraction by TRIzol (Ambion), followed by RT-PCR (described below). PCR products were analyzed on 2% agarose gels and quantified (GeneTools software; Syngene). As size markers, the sedimentation of ribosomal RNAs was analyzed (Agilent 2100 Bioanalyzer; RNA 6000 Nano Kit, Agilent).

### Polysome fractionation

HeLa cells were treated with cycloheximide (100 μg/ml) for 10 min or puromycin (250 μg/ml) for 30 min, washed once with ice-cold PBS and lysed for 10 min on ice in gradient-lysis buffer (5 mM Tris-HCl pH 7.4, 1.5 mM KCl, 5 mM MgCl_2_, 0.5% Triton X-100, 0.5% deoxycholate, Invitrogen RNaseOut, Fermentas ProteoBlock), containing cycloheximide or puromycin. After centrifugation (10 min at 20,000 × *g*), the cytoplasmic lysates were loaded onto 10–50% (w/v, 12 ml) linear sucrose gradients (20 mM Tris-HCl pH 7.4, 150 mM NaCl, 5 mM MgCl_2_, 1 mM DTT, and 100 μg/ml cycloheximide, prepared with the BioComp gradient master). Gradients were centrifuged for 3 h at 35,000 rpm in a Beckman centrifuge (SW40Ti rotor), followed by fractionation (12 × 1ml), using the Brandel tube piercer (ISCO) in combination with an ÄKTA purifier (GE Healthcare). RNA was isolated from 500 μl of the fractions (#1–11) by phenol/chloroform extraction (Roth), followed by RT-PCR (described below).

### IMP3 iCLIP

The iCLIP experiment was performed with HepG2 cells. For immunoprecipitation, IMP3-specific polyclonal antibodies (Millipore) were used, for the negative control the antibody was omitted. For validation of immunoprecipitation by Western blotting, an IMP3-specific monoclonal antibody (E2, Santa Cruz) was used. Sequencing was performed on an Illumina MiSeq instrument (75 bp single-end reads). For details on iCLIP experimental procedures and data analysis, see Rossbach *et al*.^27^ and references therein.

### Identification of IMP3-associated circRNPs

#### RNA co-immunoprecipitation

Cell lysates were prepared in RIPA buffer (50 mM Tris-HCl pH 7.4, 150 mM NaCl, 5 mM EDTA, 1% NP-40, 0.1% SDS) and subsequently cleared by centrifugation as well as by pre-incubation with protein-G dynabeads (Life technologies) without antibodies. Antibody binding was performed o/n at 4 °C, using a polyclonal IMP3 antibody (Millipore) and as mock control, FLAG-antibody (Sigma-Aldrich). Bead capturing was carried out for one hour at room temperature with protein-G dynabeads, and protein-RNA complexes were washed five times with 500 μl washing buffer (50 mM Tris-HCl pH 7.4, 150/300/600 mM NaCl, 0.05% Tween-20), increasing the stringency during the washing steps. RNA from the input (5%) and from the immunoprecipitated (IP) fractions was extracted by TRIzol (Ambion), followed by RQ1 DNase (Promega) digestion and ethanol precipitation. cDNA synthesis and RT-PCR assays are described below. RNA from co-IP experiments (mock-IP and IMP3-IP) was used for cDNA library preparation, using the TruSeq stranded total RNA sample preparation kit (Illumina) according to the manufacturer’s protocol starting with RNA fragmentation. Libraries were sequenced on a HiSeq instrument (paired-end, 2 × 100 bp, Illumina). Immunoprecipitation from gradient fractions was done as described above, starting with 100 μl of glycerol gradient fractions. The IP was carried out with a monoclonal IMP3 antibody and a monoclonal eIF4E antibody as mock control (Santa Cruz Biotechnology).

#### RNA-seq data analysis

Sequence reads were aligned to the human genome sequence (hg19) using STAR, an ultrafast universal RNA-seq aligner with chimeric alignment options^28^. Chimeric mapped reads were selected as circRNA-specific junction reads by applying four additional criteria:

1. Sequence read map to the same chromosome and the same strand, with the two sequence segments mapping to the genomic region in reverse order.
2. The overhang spanning the “back-spliced” junction is ≥12 nts.
3. The alignment score of the chimeric mapped reads (using column 14:aS of Standard SAM attributes) must be greater (>2) than the linear alignment with genomic sequences and annotated transcripts (Comprehensive Gene Annotation Set from GENCODE Version 19).
4. Both 5′ and 3′ splice sites are either annotated or conform to canonical splice sites. The circRNA abundance was predicted on the basis of circRNA specific junction read counts.

### Validation of IMP3-associated circRNAs

#### RNase R treatment

HepG2 total RNA (2 μg) was treated with or without RNase R (2.5 U/μg, Epicentre) for 20 min at 37 °C. RNA was phenolized, ethanol-precipitated, and 20% were used for RT-PCR as described below.

#### RT-PCR

RNA from gradient fractions or immunoprecipitated RNA was extracted by TRIzol (Ambion). cDNA was prepared by reverse transcription of 1 μg total RNA (RNase R assay), 10% of RNA from gradient fractions, or 10% of the coimmunoprecipitated RNAs (input/IP), using the qScript flex cDNA synthesis kit (Quanta) and random hexamer primers. Circular isoforms were PCR-amplified with divergent primers detecting the circRNA junction and linear isoforms with primers detecting the canonical splice junction downstream of the circRNA producing exons (for primer design, see ref. 29). PCR products were analyzed on 2% agarose gels (for primer sequences, see Supplementary Table S2).

Real-time PCR was carried out, using PerfeCTa SYBR Green Fast-Mix (Quanta) and an Eppendorf realplex^2^ thermocycler. Primer efficiencies were determined by four serial dilutions of cDNA derived from total RNA (R^2^ = 0,99, slope = −3.46 to −4.15). The fraction of bound target RNAs in co-IP assays was calculated from technical triplicates by the ΔΔC_t_-method, with each target normalized to the corresponding input fraction (results represented as percent of the input). Biological replicates were used to calculate standard deviations.

#### Western Blotting

Western blotting was performed with 1% of glycerol gradient fractions and a polyclonal IMP3 antibody (Millipore), which was secondarily detected with an anti-rabbit antibody.

### SELEX-seq and motif analysis of IMP3-RNA binding

#### Protein expression and purification

The IMP3 (IGF2BP3) open reading frame was PCR-amplified with IMP3_fwd/IMP3_TEV_His_rev primers, including His-tag and TEV-cleavage site, and cloned (*Eco*RI, *Xho*I) into the pGEX-6P2 expression vector (GE Healthcare). The expression of the GST-IMP3-TEV-His fusion protein was induced by IPTG (1 mM) in *E. coli BL21*, followed by a two-step purification. Cells were lysed in His lysis and washing buffer (50 mM NaH_2_PO_4_ pH 8.0, 2 M NaCl, 50 mM imidazole, 10 mM 2-mercaptoethanol, 10% glycerol, 2% Triton X-100) by sonication (three times 20 sec). The fusion protein was purified from cell lysate by incubation with Ni-NTA agarose (Qiagen) and subsequent elution (50 mM NaH_2_PO_4_ pH 8.0, 300 mM NaCl, 250 mM imidazole). The His tag was cleaved off (AcTEV-protease, 4 °C o/n, Life Technologies), and the remaining protein was purified in a second step via the GST tag (glutathione-Sepharose beads, GE Healthcare). SELEX selections were carried out with the fusion protein bound to glutathione-Sepharose.

#### SELEX (systematic evolution of ligands by exponential enrichment)

An RNA pool with a degenerated sequence of 20 nucleotides was prepared by T7 transcription, using as a template annealed SLX-N20 and T7-fw oligonucleotides (T7 High-Yield Kit, NEB). The selection was performed in a total volume of 200 μl (10 mM Tris-HCl pH 7.5, 100 mM KCl, 2.5 mM MgCl_2_, 0.1% Triton X-100, Roche protease inhibitor) with 40 pmol GST-IMP3 or GST (as negative control) bound to pre-blocked glutathione-Sepharose beads (GE Healthcare) and 4 nmol SLX-N20 transcript. After a 20 min incubation at room temperature, the samples were washed three times with 1 ml of washing buffer (10 mM Tris-HCl pH 7.5, 100/300/600 mM KCl, 2.5 mM MgCl_2_, 0.1% Triton X-100), treated with proteinase K (Roth), phenol/chloroform extracted, and ethanol precipitated. The stringency of washing steps was increased during the rounds of selection (R1: 3 × 100 mM ; R2: 2 × 100 mM, 1 × 300 mM ; R3: 1 × 100 mM, 2 × 300 mM ; R4: 1 × 300 mM and 2 × 600 mM KCl washing buffer).

Selected RNAs were reverse-transcribed (qScript Flex cDNA Synthesis Kit, Quanta), using the SLX_RT reverse primer, followed by PCR amplification with SLX_RT and SLX_T7-fw primers (16 cycles). Transcripts for the next round of selection were produced by *in vitro* transcription. After four rounds of selection, RNA aliquots from each round and from the fourth round of GST selection were used for barcoding by reverse transcription with the SLX_R13-16 (GST-IMP3) and SLX_R18 (GST) reverse primers. cDNA libraries were amplified by PCR (17 cycles; SLX_Sol-5xN_fwd and SLX_Sol_rev). All libraries were pooled in equal amounts and purified by Caliper (XT DNA 750 assay kit, Perkin-Elmer). The final library pool was subjected to high-throughput sequencing on a MiSeq instrument (single-read 100 bp, Illumina). For primer sequences, see Supplementary Table S2.

#### SELEX-seq data analysis of RNA binding of IMP3 protein

Sequence reads were first sample-barcode sorted, trimmed by PCR primer sequences on both ends, and further random-barcode filtered to obtain 18- to 20-nt sequence tags of the enriched RNA pools (numbers of filtered sequence tags given in Fig. 5A). The numbers of filtered sequence tags (from each SELEX round) containing either of the 256 or 4096 possible tetramer or hexamer motifs, respectively, was summarized, and the z-score values were calculated for enrichment of each motif.

#### IMP3 non-target circRNA group

To derive a group of circRNAs well-expressed in HepG2 cells, RNA-seq data from HepG2 whole cells [poly(A)minus selection; generated by ENCODE Consortium Long RNA-seq] were analyzed. The expression of circRNAs was determined by circRNA-specific junction counts. Based on three criteria (circular-junction counts ≥10; derived from exons of protein-coding genes; not detected in our IMP3 IP experiment), 117 circRNAs were selected as “IMP3 non-target group”.

## Additional Information

**How to cite this article**: Schneider, T. *et al*. CircRNA-protein complexes: IMP3 protein component defines subfamily of circRNPs. *Sci. Rep.* **6**, 31313; doi: 10.1038/srep31313 (2016).

## Supplementary Material

Supplementary Information
