# Supplementary material for: CircRNA-protein complexes: IMP3 protein component defines subfamily of circRNPs
Source: Sci Rep. 2016 Aug 11;6:31313. doi: 10.1038/srep31313 (PMC4980667; doi:10.1038/srep31313)
Supplement: Supplementary Information [file srep31313-s1.pdf]

SUPPLEMENT

Suppl. Figure S1

Suppl. Figure S2

Suppl. Table S1

Suppl. Table S2

CircRNA-protein complexes:
IMP3 protein component defines subfamily of circRNPs

Tim Schneider [1], Lee-Hsueh Hung [1], Silke Schreiner [1], Stefan Starke [1], Heinrich Eckhof [1], Oliver Rossbach [1], Stefan Reich [2], Jan Medenbach [2] & Albrecht Bindereif [1,*]

[1] Institute of Biochemistry, University of Giessen, Heinrich-Buff-Ring 58, D-35392 Giessen, Germany

[2] Biochemistry I, University of Regensburg, Universitätsstrasse 31, D-93053 Regensburg, Germany

* Corresponding author. Tel: +49(0)641-9935420; Fax: +49(0)641-9935419; E-mail: Albrecht.Bindereif@chemie.bio.uni-giessen.de

Legends to

Supplementary Figure S1.
CircRNA-protein complexes in mammalian cells:
comparing S100, cytoplasmic, and nuclear extracts
(**A/B**) Sedimentation profiles of circRNPs from HeLa cells. Cytoplasmic S100 extract (S100), cytoplasmic extract (CE), nuclear extract (NE), as well as free RNA (RNA; prepared from S100 extract), and S100 and CE extracts after proteinase K treatment (S100 extract+PK; CE+PK) were fractionated by glycerol gradient centrifugation (#1-22 from top to bottom; the last fraction containing the resuspended pellet). The circRNA distributions across the gradients were determined by RT-PCR: (**A**) for three abundant circRNAs (CAMSAP1, GLIS3, and HIPK3; related to **Fig. 1A**), and (**B**) for two IMP3-associated circRNAs (CDYL and NFATC3; related to **Fig. 3C**). The positions of ribosomal RNA size markers are indicated (5S, 18S, and 28S), and the brackets mark the circRNA vs. circRNP peak fractions from cytoplasmic S100 and cytoplasmic extract (CE). The circRNP distribution in nuclear extract (NE) was detectable only for CAMSAP1, GLIS3, and HIPK3 circRNAs. The sedimentation of IMP3 protein in cytoplasmic S100 extract (S100 extract) and cytoplasmic extract (CE) was visualized by Western Blot.

Supplementary Figure S2.
iCLIP analysis of IMP3-RNA interactions in HepG2 cells
A. Summary of iCLIP sequence reads (left part) and distribution of barcode-filtered crosslink tag counts in genome (right part).
B. Barcode-filtered crosslink tag counts, based on our IMP3 iCLIP-Seq data, are represented for these genes: FTL (positive linear mRNA control); CAMSAP1 (negative circRNA control); CDYL, NFATC3, and ANKRD17 (IMP3 circRNA targets; with the circularizing exons highlighted).

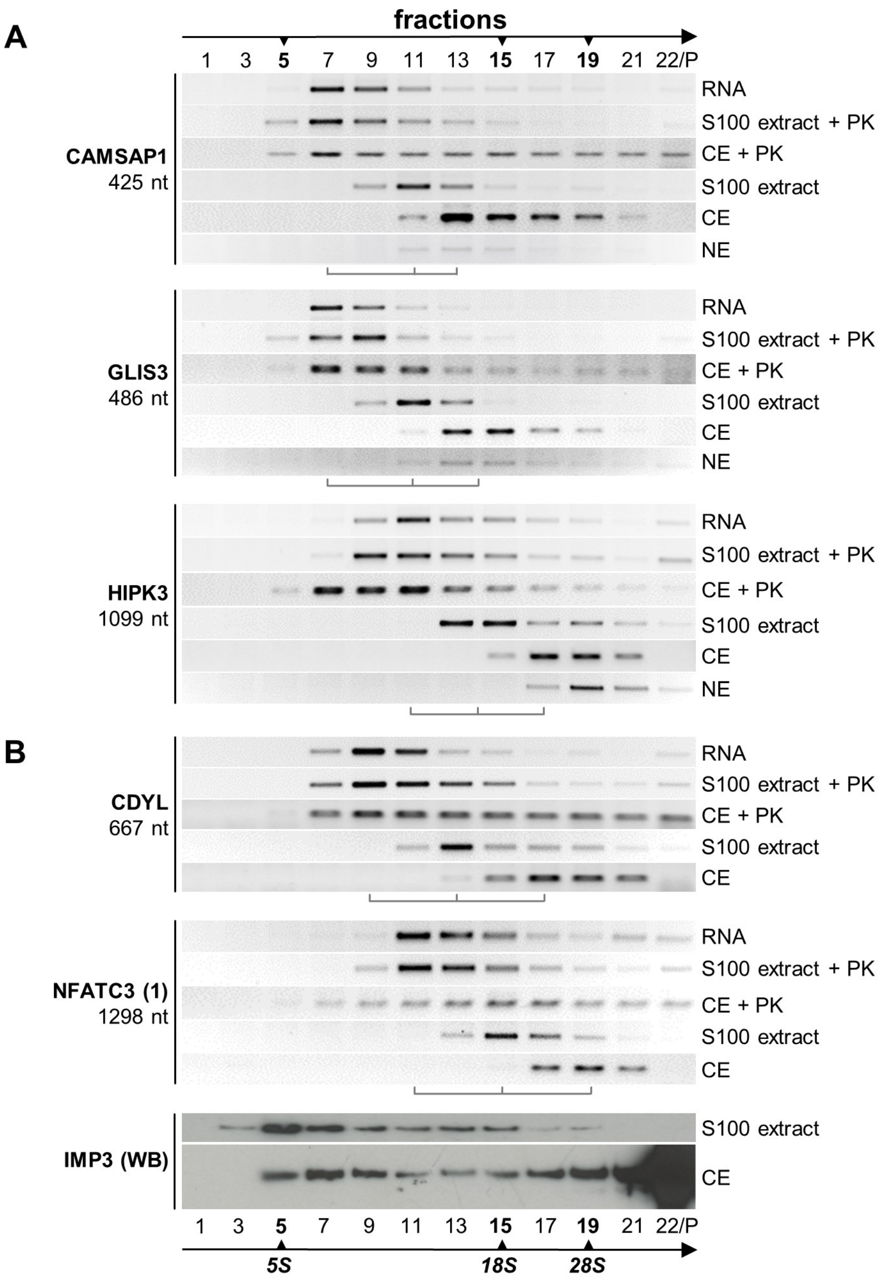

A
B
fractions
1
3
5
7
9
11
13
15
17
19
21
22/P
CAMSAP1
425 nt
GLIS3
486 nt
HIPK3
1099 nt
CDYL
667 nt
NFATC3 (1)
1298 nt
IMP3 (WB)
RNA
S100 extract + PK
CE + PK
S100 extract
CE
NE
5S
18S
28S

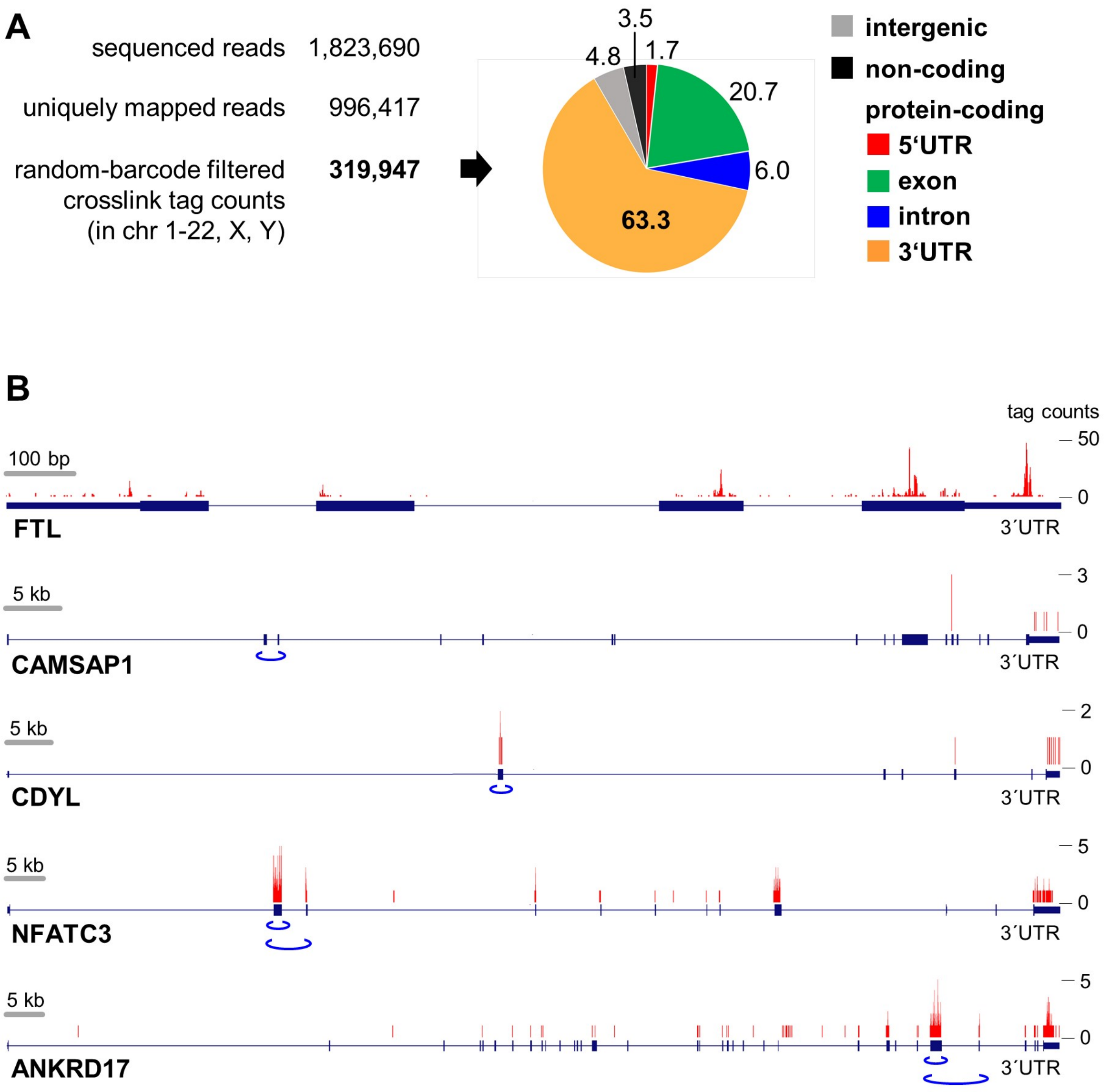
A
sequenced reads 1,823,690
uniquely mapped reads 996,417
random-barcode filtered crosslink tag counts (in chr 1-22, X, Y) 319,947
3.5
4.8
1.7
20.7
6.0
63.3
intergenic
non-coding
protein-coding
5'UTR
exon
intron
3'UTR
B
tag counts
100 bp
50
0
FTL
3´UTR
5 kb
3
0
CAMSAP1
3´UTR
5 kb
2
0
CDYL
3´UTR
5 kb
5
0
NFATC3
3´UTR
5 kb
5
0
ANKRD17
3´UTR

Supplementary Table S1. IMP3-associated circRNAs (≥ 30 circRNA-specific junction read counts in at least one cell line)

***** total number of top 10 enriched tetramers (SELEX-based)

	chromosome_position				genomic	circRNA	circRNA-specific junction counts					motif
gene_symbol	strand		start	end	length(nts)	length(nts)	HepG2	PANC1	PATU	transcript_id	circular exons	counts*
ARID1A	+	chr1	27056142	27059283	3142	783	21	61	54	ENST00000457599.2	e2-e4	34
ANKRD17(2)	-	chr4	73956384	73958017	1634	1634	81	16	251	ENST00000358602.4	e29	119
ANKRD17(1)	-	chr4	73950966	73958017	7052	1832	43	6	63	ENST00000358602.4	e29,e30	134
ATXN1	-	chr6	16326625	16328701	2077	2077	35	17	7	ENST00000436367.1	e7	81
C11orf30	+	chr11	76174865	76183884	9020	537	5	7	49	ENST00000533248.1	e7,e8	55
CCAR1	+	chr10	70497602	70502326	4725	272	1	2	33	ENST00000541012.1	e4-e6	26
CDC73	+	chr1	193172925	193181607	8683	182	1	4	32	ENST00000367435.3	e11-e13	10
CDYL	+	chr6	4891947	4892613	667	667	53	33	72	ENST00000449732.2	e2	33
CYP24A1	-	chr20	52773708	52788209	14502	1106	35	2	85	ENST00000216862.3	e3-e11	48
DROSHA	-	chr5	31421379	31424578	3200	309	1	9	40	ENST00000513349.1	e27-e29	16
ELK4	-	chr1	205585606	205593019	7414	1373	11	24	50	ENST00000357992.4	e2-e5	71
FAT1	-	chr4	187627717	187630999	3283	3283	61	11	30	ENST00000441802.2	e2	165
FIP1L1(1)	+	chr4	54280782	54294350	13569	359	3	0	45	ENST00000358575.5	e10-e12	15
FIP1L1(2)	+	chr4	54292039	54310270	18232	389	5	8	57	ENST00000358575.5	e11-e15	18
FNDC3B	+	chr3	171965323	171969331	4009	526	75	139	114	ENST00000416957.1	e5,e6	35
FOXP1	-	chr3	71090479	71102924	12446	587	32	1	5	ENST00000468577.1	e3-e6	37
NEIL3(2)	+	chr4	178274462	178274882	421	421	2	0	70	ENST00000264596.3	e8	29
NEIL3(1)	+	chr4	178274462	178281831	7370	596	0	6	53	ENST00000264596.3	e8,e9	37
NFATC3(2)	+	chr16	68155890	68157024	1135	1135	109	31	298	ENST00000575270.1	e2	57
NFATC3(1)	+	chr16	68155890	68160513	4624	1298	39	9	62	ENST00000575270.1	e2,e3	63
PHC3(1)	-	chr3	169840379	169847340	6962	1023	8	18	168	ENST00000494943.1	e8,e9	60
PHC3(2)	-	chr3	169863211	169867032	3822	258	19	21	286	ENST00000494943.1	e5,e6	12
PHC3(3)	-	chr3	169854207	169867032	12826	505	17	29	398	ENST00000494943.1	e5-e7	34
PTPRA	+	chr20	2944918	2945848	931	421	6	10	67	ENST00000425918.2	e3,e4	27
R3HDM1	+	chr2	136432902	136437894	4993	307	3	13	50	ENST00000409606.1	e19,e20	22
SAMD4A	+	chr14	55168780	55169298	519	519	5	1	31	ENST00000554335.1	e3	33
SPECC1	+	chr17	20107646	20109225	1580	1580	15	4	63	ENST00000395527.4	e4	72
TAB2(1)	+	chr6	149699154	149700654	1501	1501	6	3	37	ENST00000392282.1	e3	111
TAB2(2)	+	chr6	149691045	149700654	9610	1692	10	8	62	ENST00000392282.1	e2,e3	119
TEX10	-	chr9	103082547	103111654	29108	2211	10	1	37	ENST00000374902.4	e2-11	112
UBAP2(1)	-	chr9	33948372	33953472	5101	404	3	25	84	ENST00000418786.2	e10,e11	31
UBAP2(2)	-	chr9	33948372	33956144	7773	472	6	29	64	ENST00000418786.2	e9-e11	38
UBAP2(3)	-	chr9	33941647	33948585	6939	873	6	17	42	ENST00000418786.2	e11-e14	61
VWA8	-	chr13	42385361	42393522	8162	363	3	3	38	ENST00000281496.6	e15-e17	29

Supplementary Table S2. List of oligonucleotides.

Gene	Primer name	Sequence	Method
**Gradient sedimentation analysis**			
AFAP1	AFAP1_circ_fwd	GAAGGACCATGCTCAGAAGC	RT-PCR
	AFAP1_circ_rev	CACTGCCTTTTTCTCCCTGA	
ASAP1	ASAP1_circ_fwd	GACTACAACTCGCCCACCAC	RT-PCR
	ASAP1_circ_rev	TGAAACCATGCCTCAGTGAA	
ASPH	ASPH_circ_fwd	TCAGAGGTGCTTCAAGGAAAA	RT-PCR
	ASPH_circ_rev	CCAGCAATGCAATCACCATA	
ASXL1	ASXL1_circ_fwd	GCATGCCTCAATGCTATGCT	RT-PCR
	ASXL1_circ_rev	GGGGAAGTCCCACTTCTCAT	
CAMSAP1	CAMSAP1_circ_fwd	CCCTGATGATGGCCTACACT	RT-PCR/RT-qPCR
	CAMSAP1_circ_rev	TGTGCTCCTGCTCATACTGG	
CDYL2	CDYL2_circ_fwd	ATGGGTGAATGTGACGTGAA	RT-PCR
	CDYL2_circ_rev	TCCTCACAGTGCAAGAGGTG	
CORO1C	CORO1C_circ_fwd	ATGGGTTACATGCCCAAGAG	RT-PCR
	CORO1C_circ_rev	ACTGCTGTCACCCTTTCCAC	
CRKL	CRKL_circ_fwd	TCATGCATACGCTCAACCTC	RT-PCR
	CRKL_circ_rev	GGTTGGGTGCTGAGACAGAT	
EPHB4	EPHB4_circ_fwd	CCCGTCATGATTCTCACAGA	RT-PCR
	EPHB4_circ_rev	TTGATTGCCACACAGCTCTC	
FARSA	FARSA_circ_fwd	TCCGACAGATCTTCCTGGAG	RT-PCR
	FARSA_circ_rev	CTGGGCTCAGCTCTGTCTCT	
GLIS3	GLIS3_circ_fwd	GTTTGGAAGCCCTTTTCCTC	RT-PCR
	GLIS3_circ_rev	GATGTCCGGTGGAGACTCAT	
GSE1	GSE1_circ_fwd	CCAGCTTTGCCGCCGCGCTG	RT-PCR
	GSE1_circ_rev	GTGGAAAGCATCCCTAGCG	
HIPK3	HIPK3_circ_fwd	TCGGCCAGTCATGTATCAAA	RT-PCR
	HIPK3_circ_rev	CCCTTAGTGGGAGGATGAGA	
LPAR1	LPAR1_circ_fwd	TGGCTGCCATCTCTACTTCC	RT-PCR
	LPAR1_circ_rev	CGAAGTCATGCTAGGAGAAGC	
SH3PXD2A	SH3PXD2A_circ_fwd	GGGATGACTCCGACATCAAC	RT-PCR
	SH3PXD2A_circ_rev	CTCAGCTCCGAGTTCTCCTG	
**Identification of IMP3-associated circRNAs**			
FTL	FTL_linE3-4_fwd	ATCTTCATGCCCTGGGTTCT	RT-PCR/RT-qPCR
	FTL_linE3-4_rev	GAGGTTGGTCAGGTGGTCA	
ANKRD17	ANKRD17_linE31-32_fwd	TCTGCACGTATCAGGCAAAC	RT-PCR/RT-qPCR
	ANKRD17_linE31-32_rev	ATTTCCCATCCCAGGGTTAC	
	ANKRD17_circE29-30_fwd (1)	CAGGAGGTCAGATGTACGGA	
	ANKRD17_circE29-30_rev (1)	TCTTGTTGATTCAGTGCCACC	
ARID1A	ARID1A_linE5-6_fwd	AGTGTTGCTCAGTCTCGCTC	RT-PCR
	ARID1A_linE5-6_rev	CGATCTTGGGCAATGCTTGA	
	ARID1A_circE2-4_fwd	CCAGTAAGGGAGGGCAAGAA	
	ARID1A_circE2-4_rev	TGCTGCGAGTATGGGTTAGT	

Supplementary Table S2. List of oligonucleotides.

Gene	Primer name	Sequence	Method
PHC3	PHC3_linE10-11_fwd	TCAGTGTGTGTTCAGCCAGA	RT-PCR
	PHC3_linE10-11_rev	CTGAAGCACTGACTGATGCC	
	PHC3_circE8-9_fwd (1)	ACAGTGGGGAGAGGAGAAGA	
	PHC3_circE8-9_rev (1)	TGGGAAACTTTGGAAGGTGG	
CDYL	CDYL_linE5-6_fwd	GGCTTCACCCACATCTTGTT	RT-PCR/RT-qPCR
	CDYL_linE5-6 _rev	GTACCAGCTTGCTGTCATCG	
	CDYL_circE4_fwd	CAGGCTTAGCTGTTAACGGG	
	CDYL_circE4_rev	TGTCATAGCCTTTCCACCGA	
ELK4	ELK4_linE4-5_fwd	ACTCTCAGTCCTGTTGCTCC	RT-PCR
	ELK4_linE4-5_rev	ACAGAGTGAATGGCCCATGA	
	ELK4_circE2-5_fwd	GTGGAATGAGAGAACCGAGGA	
	ELK4_circE2-5_rev	GTGGAATGAGAGAACCGAGGA	
FNDC3B	FNDC3B_linE7-8_fwd	GTTGGAAGTAAAGAGGGTGCA	RT-PCR
	FNDC3B_linE7-8_rev	CCACCTCGTAACTGTAGGGG	
	FNDC3B_circE5-6_fwd	CGGTAGTGGTCCCGGAATTA	
	FNDC3B_circE5-6_rev	TGGGTGACATCATGGGAACT	
NEIL3	NEIL3_lin6-7_fwd	GTCCTAATTGTGGTCAGTGCC	RT-PCR
	NEIL3_lin6-7_rev	CCACCCTGCTAGATGTCCAA	
	NEIL3_circE8_fwd (2)	AGCTGCAACCCTGGATATTCT	
	NEIL3_circE8_rev (2)	AGAGTTGTAGTTCCAAATGCAGT	
NFATC3	NFATC3_linE4-5_fwd	CGACCTCATGCATTTTACCA	RT-PCR/RT-qPCR
	NFATC3_linE4-5_rev	TCTGAATTGCGGAGTTTCAA	
	NFATC3_circE2_fwd (1+2)	ACCTGGAGCAAACCAAAGCC	
	NFATC3_circE2_rev (2)	TGTGGTAAGCAAAGTGGTGTG	
	NFATC3_circE3_fwd (1)	TGAAACTGAAGGTAGCCGAGG	
**SELEX-Seq and motif analysis of IMP3 RNA binding**			
IMP3	IMP3_fwd	TGATGAACAAACTGTATATCGGAA	Cloning
	IMP3_TEV_His_rev	TGACCTCGAGTTAATGATGATGATGATGATGACCTTGAAAATAAAATTTTCCTTCCGTCTTGACTGAGGTG	
Random RNA pool	SLX_N20	GCGTCTCGAGCGTAGTTANNNNNNNNNNNNNNNNNNNNAGTCGGCATCTTGGTACCTATAGTGAGTCGTATTA	SELEX
	T7_fwd	TAATACGACTCACTATAGGG	
	SLX_RT	GCGTCTCGAGCGTAGTTA	
	SLX_T7_fwd	TAATACGACTCACTATAGGGTACCAAGATGCCGACT	
Selection round 1	SLX_R13	CAAGCAGAAGACGGCATACGAGATCGGTCTCGGCATTCCTGNNATATNNNGCGTCTCGAGCGTAGTTA	Library preparation
Selection round 2	SLX_R14	CAAGCAGAAGACGGCATACGAGATCGGTCTCGGCATTCCTGNNGGCANNNGCGTCTCGAGCGTAGTTA	
Selection round 3	SLX_R15	CAAGCAGAAGACGGCATACGAGATCGGTCTCGGCATTCCTGNNTGTANNNGCGTCTCGAGCGTAGTTA	
Selection round 4	SLX_R16	CAAGCAGAAGACGGCATACGAGATCGGTCTCGGCATTCCTGNNGGACNNNGCGTCTCGAGCGTAGTTA	
Selection round 4 (GST)	SLX_R18	CAAGCAGAAGACGGCATACGAGATCGGTCTCGGCATTCCTGNNGCGTNNNGCGTCTCGAGCGTAGTTA	
	SLX_Sol-5xN_fwd	AATGATACGGCGACGTCCGAGATCTACACTCTTTCCCTACACGACGCTCTTCCGATCTNNNNNGGTACCAAGATGCCGACT	
	SLX_Sol_rev	CAAGCAGAAGACGGCATACG	
